# Lower Exposure and Faster Clearance of Bevacizumab in Gastric Cancer and the Impact of Patient Variables: Analysis of Individual Data from AVAGAST Phase III Trial

**DOI:** 10.1208/s12248-014-9631-6

**Published:** 2014-06-19

**Authors:** Kelong Han, Jin Jin, Mauricio Maia, John Lowe, Martina A. Sersch, David E. Allison

**Affiliations:** Genentech, Inc., 1 DNA Way, South San Francisco, California 94080 USA

**Keywords:** advanced gastric cancer, bevacizumab, ethnicity, NONMEM, pharmacokinetics

## Abstract

Altered pharmacokinetics of antibody drugs has been reported in advanced gastric cancer (AGC). We aim to evaluate bevacizumab pharmacokinetics in AGC from the Phase III trial (AVAGAST), and explore the influence of patient variables. Bevacizumab concentrations (Cp) were measured in plasma samples taken following disease progression from 162 patients (7.5 mg/kg every 3 weeks). Predicted Cp [median and 90% prediction interval] was simulated using the population pharmacokinetic model established for other cancers (PPK model) and compared to observed Cp. Bevacizumab clearance was estimated using NONMEM and compared between subgroups. Patient characteristics of AGC are similar to other cancers except for lower body weight despite higher percentage of males. Eighty-five percent of observed Cp was below the median predicted Cp and 38% below the lower boundary of the 90% prediction interval. Median bevacizumab clearance in AGC was 4.5 *versus* 3 mL/day/kg in other cancers. Bevacizumab clearance was significantly faster (*p* < 0.05) in patients without gastrectomy (*n* = 42) or lower albumin. Clearance appeared to be faster in patients with lower total protein, higher ECOG scores, more metastatic sites, and poorer response. No significant difference in bevacizumab concentrations and clearance was observed between Asian and Non-Asian patients. AGC patients exhibited significantly lower bevacizumab exposure due to an approximate 50% increase in clearance *versus* other cancers. Bevacizumab is cleared faster in patients without prior gastrectomy. No significant difference in bevacizumab pharmacokinetics was observed between Asian and Non-Asian patients. The underlying mechanism for faster bevacizumab clearance in AGC is unknown and warrants further research.

**Electronic supplementary material:**

The online version of this article (doi:10.1208/s12248-014-9631-6) contains supplementary material, which is available to authorized users.

## INTRODUCTION

Bevacizumab (Avastin®, Genentech Inc., South San Francisco, CA) is a humanized monoclonal immunoglobulin G (IgG) antibody that specifically binds and inactivates vascular endothelial growth factor A (VEGF-A), a key isoform of VEGF involved in angiogenesis, and a well-characterized pro-angiogenic factor (1). Bevacizumab causes inhibition of tumor angiogenesis by blocking VEGF-A from binding to its receptors and leads to tumor growth inhibition. Bevacizumab, in combination with standard chemotherapy, was approved to treat metastatic colorectal cancer, non-small cell lung cancer, breast cancer, glioblastoma, renal cell carcinoma, and ovarian cancer (2–7). Furthermore, bevacizumab has been or is being investigated for pancreatic cancer, hepatic cancer, prostate cancer, soft-tissue sarcomas, and gastric cancer (GC) (8–13).

Gastric cancer is the fourth most common malignancy in the world and the third most frequent cancer related death after lung cancer and liver cancer (http://globocan.iarc.fr/). Bevacizumab was evaluated in first-line advanced gastric cancer (AGC) in a double-blind randomized Phase III trial Avastin in Gastric Cancer (AVAGAST) with 387 patients in the bevacizumab and placebo arm, respectively (13). Both median progression-free survival (6.7 *versus* 5.3 months; hazard ratio, 0.80; *p* = 0.0037) and overall response rate (46.0% *versus* 37.4%; *p* = 0.0315) were significantly improved with bevacizumab *versus* placebo. However, the primary endpoint of overall survival was not met in the intent-to-treat population (hazard ratio, 0.87; *p* = 0.1002).

Bevacizumab pharmacokinetics is well established in phase-I, -II, and -III studies as a single agent or in combination with chemotherapy for both single- and multiple-dose administration with both rich and sparse bevacizumab serum concentration data (14–17). Bevacizumab pharmacokinetics showed dose linearity within the dose range of 1–20 mg/kg, a slow clearance, a volume of distribution consistent with limited extravascular distribution, and a terminal half life of approximately 20 days. Short-term (*e.g*., up to 30 days after dose) and long-term (up to 6 months after dose) pharmacokinetics of bevacizumab is comparable (17). A population pharmacokinetic model has been previously established in metastatic colorectal, breast, non-small cell lung, and prostate cancer, and showed that bevacizumab pharmacokinetics is consistent across these cancers (15).

However, bevacizumab pharmacokinetics has never been previously evaluated in GC. The pharmacokinetic analysis of the data from the randomized double-blind phase III trial investigating trastuzumab (Herceptin®, Genentech Inc., South San Francisco, CA) in combination with chemotherapy in AGC (ToGA) showed that the exposure of the intravenous formulation of trastuzumab was significantly lower in AGC as compared to other solid tumors at the same dose (18,19) although the cause of the altered clearance of the intravenous formulation of trastuzumab is not fully elucidated and may be multifactorial. A case–control analysis using the same data showed that patients with the lowest quartile of the trough serum concentration of the intravenous formulation of trastuzumab did not benefit in overall survival, from addition of trastuzumab treatment to chemotherapy (20). Furthermore, the pharmacokinetic analysis of the data from the randomized phase II trial investigating pertuzumab (Perjeta®, Genentech Inc., South San Francisco, CA) in combination with chemotherapy and the intravenous formulation of trastuzumab in AGC (JOSHUA) showed that the mean pertuzumab trough concentration was approximately 37% lower in AGC than that observed in breast cancer at the same dose (21). These results led to the hypothesis that the pharmacokinetic properties of antibody drugs may be altered in AGC, and the pharmacokinetic exposure in AGC may fall below the concentrations that have been proven efficacious in other solid tumors such as breast cancer. Therefore, an investigation of bevacizumab pharmacokinetics in AGC is warranted.

Pharmacokinetic evaluation of bevacizumab was not pre-specified in the AVAGAST protocol. However, plasma samples for research purposes were collected following disease progression, allowing for the opportunity to investigate bevacizumab pharmacokinetics in AGC.

Geographically, GC is concentrated in East Asia. Korea, Japan, and China account for 60% of new cases and 56% of death cases every year (http://globocan.iarc.fr/). In recent multi-regional studies in AGC (13,22,23), Eastern patient population consistently demonstrated longer overall survival in both arms compared to the Western patient population. In addition, the hazard ratios of overall survival between the treatment and control arm were different in Eastern and Western patient populations in these trials (Supplement Table 1). Based on limited pharmacokinetic data, there is no evidence that bevacizumab pharmacokinetics differ between Asian and Non-Asian patient populations. However, no direct comparison has been made. AVAGAST enrolled almost equal number of Asian (Japan and Korea) and Non-Asian patients, offering an opportunity for the first time to perform a head-to-head comparison of bevacizumab pharmacokinetics between Asian and Non-Asian AGC patients.

Our objectives were to (1) characterize bevacizumab exposure and pharmacokinetics in AGC, (2) compare bevacizumab pharmacokinetics between AGC and other solid tumors, and (3) explore the association between bevacizumab pharmacokinetics and patient variables.

## MATERIALS AND METHODS

### Studies and Data Collection

Per AVAGAST protocol (13) (ClinicalTrials.gov identifier: NCT00548548), patients with previously untreated, histologically confirmed, unresectable, locally advanced or metastatic adenocarcinoma of the stomach or gastroesophageal junction were assigned to bevacizumab or placebo in combination with cisplatin administered for six cycles plus capecitabine administered until disease progression or intolerable adverse effects. Bevacizumab was administered at 7.5 mg/kg every 3 weeks *via* intravenous infusion and was discontinued following disease progression. One plasma sample for research purposes was collected following disease progression from each patient whenever possible. Complete dosing records and patient variables (demographic, prognostic, and biochemical factors) were collected. The protocol was approved at each participating site by an independent ethics committee or institutional review board. All patients were provided written informed consent before study entry.

### Bioanalytical Assay

Bevacizumab concentrations were determined in ethylenediaminetetraacetic acid (EDTA) plasma at Genentech, Inc. using an enzyme-linked immunosorbent assay (ELISA) adapted from the existing serum ELISA for bevacizumab (24). Similar to the serum ELISA, the EDTA plasma assay uses recombinant human VEGF for capture of circulating bevacizumab onto ELISA plates, and the same polyclonal goat anti-human IgG Fc antibody conjugated to horseradish peroxidase for detection. The sample minimal dilution in both PK assays is 1:100, done in assay buffer. The assay was qualified for the testing of human plasma samples with a lower limit of quantification of 78 ng/mL. Additional qualification experiments showed that the assay accurately quantifies bevacizumab spiked into human plasma samples of individual donors, obtained from a commercial source (Bioreclamation, LLC; Baltimore, MD). Overall, the assay presented adequate accuracy, precision, dilution linearity, and specificity. The presence of VEGF in samples, at expected physiological concentrations, did not markedly interfere in the quantification of bevacizumab. In addition to following the same assay format as the serum assay, the standard curve of the plasma assay was shown to be superimposable to the standard curve of the serum assay. Furthermore, both assays were found to have comparable performance by all parameters assessed, including assay sensitivity, dilution linearity, target (VEGF) interference, and spike recovery in matrix.

### Reference PPK Model

A population pharmacokinetic analysis (reference PPK model) has been previously conducted using pooled data from 533 patients in metastatic colorectal, breast, non-small cell lung, and prostate cancer (15), and then updated with first-order conditional estimation method with η–ε interaction (FOCE INTER) method. Model details are summarized in Supplement Table 2.

### Pharmacokinetic Analysis

Visual predictive check (VPC) and Empirical Bayes estimation were performed in NONMEM 7.1.2 (ICON Development Solutions, Dublin, Ireland) using FOCE INTER method. All other analysis and graphing were performed using R 2.15.1 (25). Clinically relevant patient variables were selected *a priori* (Table I).

**Table I:** Patient Characteristics

Gastric cancer patients (*n* = 162)	
Age (years)	58 (28∼81)
Body weight (kg)	59.9 (35.7∼100.2)
Serum albumin (g/L)	39 (27∼48.3)
Total protein (g/L)	68 (49∼85.4)
ECOG score 0/1/2	47.5%:45.7%:6.8% (77:74:11)
Gender (male/female)	67.3%:32.7% (109:53)
Baseline plasma VEGF-A (pg/mL)	110.2 (20∼1,868)
Lactate dehydrogenase (U/L)	220 (50∼2,675)
Disease control rate (SD/PR/CR)	82.7% (134)
Number of metastatic sites >1	69.8% (111)
Prior gastrectomy (no/yes)	74.1% (120)
Asian	62.3% (101)
Type of gastric cancer: diffuse/intestinal/mixed/unknown	47%:39%:9%:6% (76:63:14:9)
Prior chemotherapy	6% (9)
Location of primary tumor: gastroesophageal junction/stomach	14%:86% (22:140)
Extent of disease: locally advanced/metastatic	2.5%:97.5% (4:158)
Stage IIIA/IV	2%:96% (3:156)
Liver metastases	36.4% (59)
Bone metastases	4.9% (8)
HER2-negative	82.7% (134)
Sampling day after the last dose	25 (2∼284)
Number of doses received	7 (1∼21)
Reference PPK model	
Age (years)	59 (21∼88)
Body weight (kg)	74 (49∼114)
Serum albumin (g/L)	37 (29∼44)
Total protein (g/L)	72 (62∼83)
ECOG 0:1:2	54.0%:44.2%:1.8%
Gender (male/female)	43.8%:56.2%

All values are expressed as median (range) or percentage of the patients (number of the patients)

*ECOG* Eastern Cooperative Oncology Group, *VEGF*-*A* vascular endothelial growth factor A, *SD* stable disease, *PR* partial responses, *CR* complete responses, *PPK* population pharmacokinetic

In the VPC, expected (simulated) bevacizumab pharmacokinetic concentration-*versus*-time profiles [median and 90% prediction interval (PI)] were simulated (1,500 times) using the final covariate reference PPK model and the patient variables collected in AVAGAST, and then compared to the observed bevacizumab concentrations in AVAGAST. The individual predicted bevacizumab concentration at the time of sampling was also calculated for each patient using the final covariate reference PPK model, and was compared to the observed data.

In the Empirical Bayes estimation, posterior Bayes pharmacokinetic parameter estimates were obtained by fitting the reference PPK model to the observed data with the estimation option MAXEVALS set to 0 and POSTHOC. The resulting individual bevacizumab pharmacokinetic parameter estimates were compared with those from the reference PPK model (other solid tumors), and were compared between patient subgroups stratified by patient variables (demographic, prognostic, and biochemical factors). The expected bevacizumab pharmacokinetic parameters were calculated for each patient using the final covariate reference PPK model (“expected” parameters), and compared to the estimated bevacizumab pharmacokinetic parameters based on observed data (“observed” parameters).

### Statistical Analysis

All statistical analysis and graphing were performed using R 2.15.1 (25). Log transformation was applied when the data was log-normally distributed. The relationship between pharmacokinetic parameters and continuous patient variables was examined by linear regression analysis. The relationship with dichotomous variables was evaluated using unpaired two-tailed Student’s *t* test or one-way analysis of variance. The observed and expected (simulated) concentrations or pharmacokinetic parameters in the same patient were compared using paired two-tailed Student’s *t* test. In addition, the ratios of geometric means were calculated. The 95% confidence intervals (CI) for the ratios of geometric means were obtained by back transformation.

## RESULTS

### Patient Population and Characteristics

**Fig. 1:**
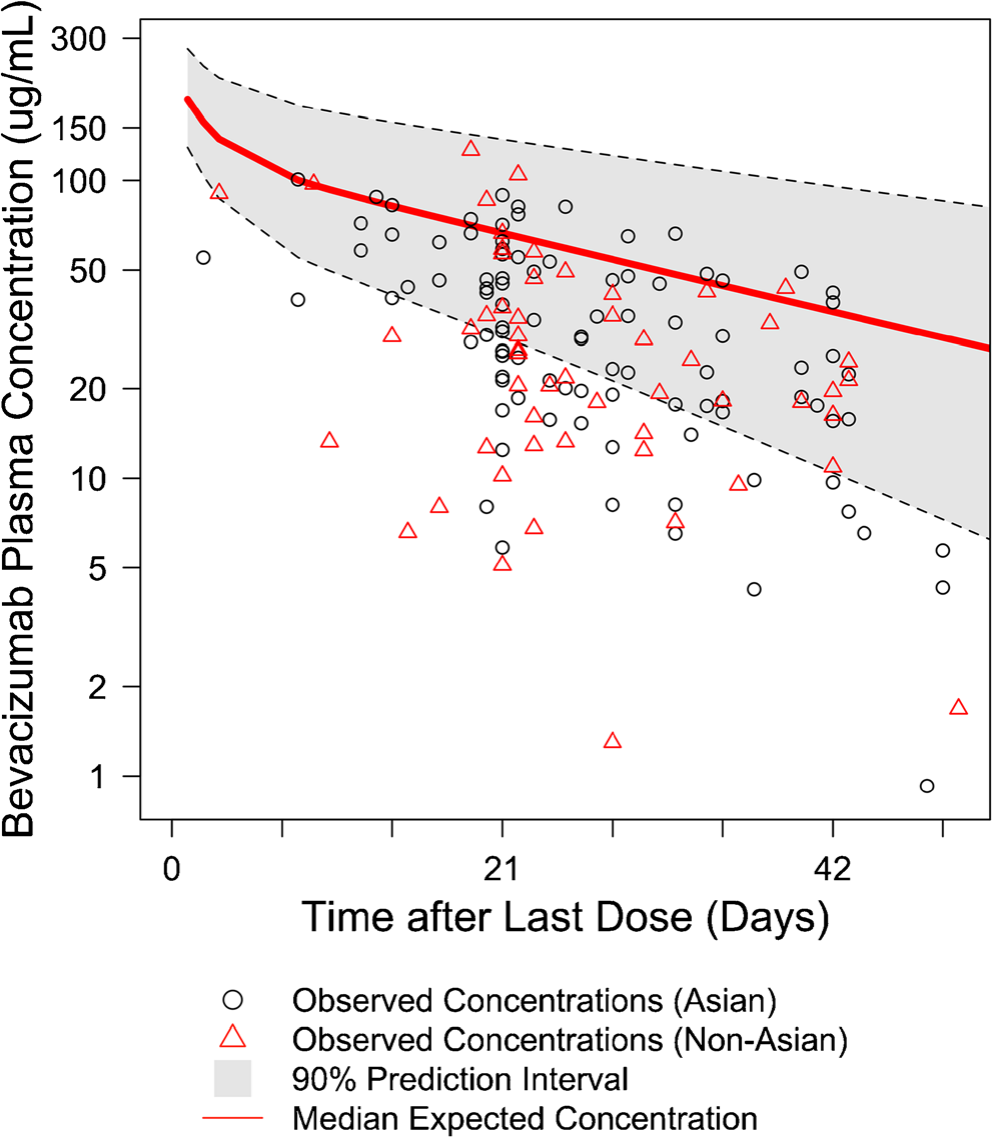
Visual predictive check (VPC) comparing observed and expected (simulated) bevacizumab concentrations. Only the 151 patients with a sampling time within 50 days after the last bevacizumab dose are shown (93.2% of the 162 evaluable patients)

A total of 182 pharmacokinetic plasma samples were able to be collected following disease progression from 182 patients in AVAGAST (47% of the patients in the bevacizumab arm). The sampling time varied from days 2 to 394 after the last dose of bevacizumab. Bevacizumab concentrations were below lower limit of quantification (LLOQ) in 20 samples, leaving 162 evaluable pharmacokinetic samples (Fig. 1a). Patient characteristics of the 162 patients were consistent with the whole population in AVAGAST (Table I). Most of the patient characteristics of the 162 patients were similar with the patient population in the reference PPK model except for substantially lower body weight (59.9 *versus* 74 kg) and higher percentage of males (67.3% *versus* 43.8%). The sampling time was within 50 days after the last bevacizumab dose in 93.2% (*n* = 151) of the 162 evaluable patients.

### Visual Predictive Check

The median number of bevacizumab doses received was 7, and the 25th and 75th percentile was 5 and 10 doses. Therefore, steady state was assumed. The observed bevacizumab concentrations were significantly below the expected (simulated) bevacizumab pharmacokinetic concentration-*versus*-time profiles [median and 90% PI] (Fig. 1a, b). Only 1 out of the 162 observed concentrations was above the upper boundary of the 90% PI, 85.2% of the observed concentrations fell below the median, and 38.3% below the lower boundary of the 90% PI.

The individual observed and expected bevacizumab concentration was compared in Fig. 1c. The observed concentrations were below the expected concentrations in 82% of the patients (*n* = 133). The observed bevacizumab concentrations are significantly lower than expected by 47% on average (*p* < 0.0001, paired *t* test). The ratio of geometric means of observed concentrations and expected contractions was 0.52 (95% CI = 0.46 to 0.60).

### Empirical Bayes Estimation

The individual pharmacokinetic parameters were estimated with the covariate for chemotherapy set to 1. Due to the significant difference in body weight and gender percentage between these 162 AGC patients and the reference patient population (other solid tumors), weight normalization was applied whenever appropriate. Median weight-normalized clearance values in AGC patients (4.5 mL/day/kg) were 50% higher than other cancer types (3 mL/day/kg).

**Fig. 2:**
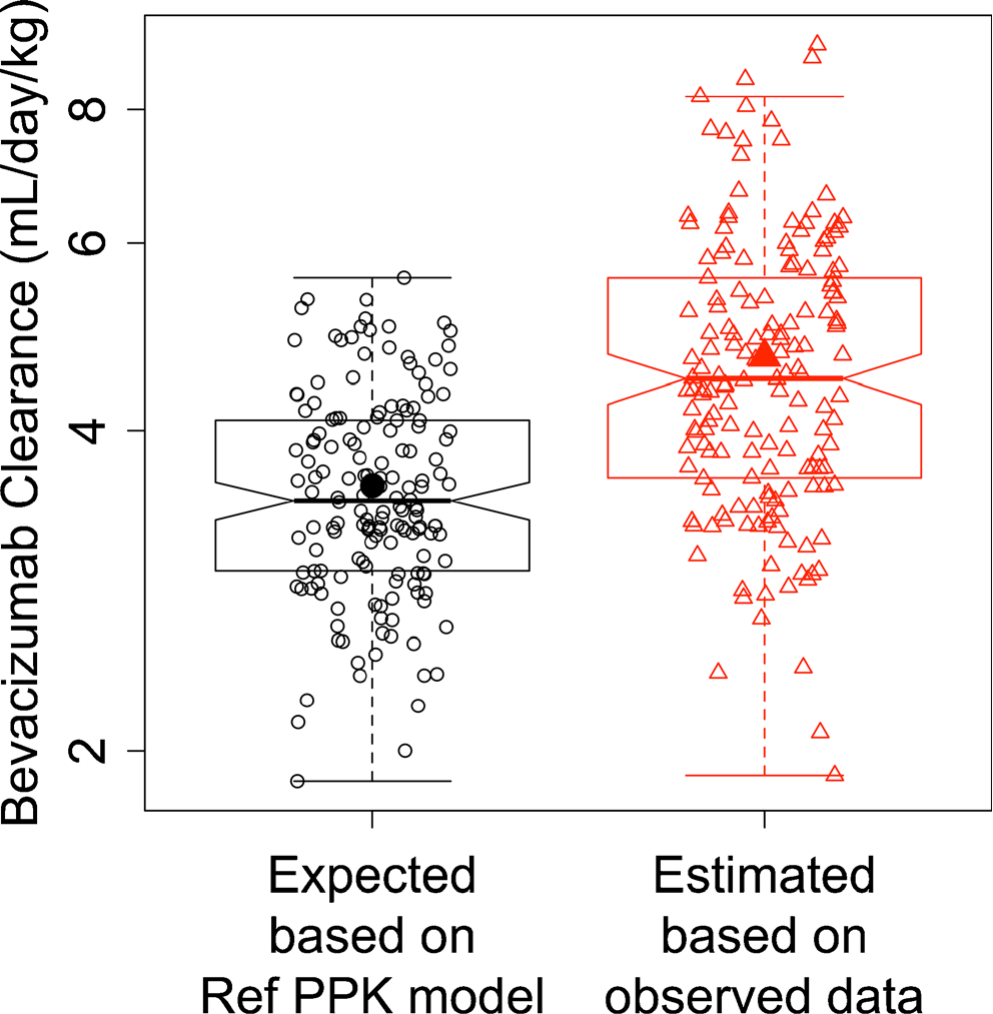
Comparison of individual bevacizumab clearance that is predicted (expected) based on the reference population pharmacokinetic model and estimated based on observed data *via* Empirical Bayes estimation. *Solid symbols* represent the mean. *Hollow symbols* represent individual data. Ref: reference

Figure 2a compared the individual clearance that was estimated based on observed data (noted as “observed”) and expected based on the reference PPK model (noted as “expected”). The “observed” clearance values are significantly higher than the “expected” (*p* < 0.0001, paired *t* test), and is above expected in 82% of the patients (Fig. 2b). The ratio of geometric means of “expected” clearance and “observed” clearance values was 0.77 (95% CI = 0.74 to 0.81).

### Impact of Patient Variables on Bevacizumab Exposure and Clearance

**Fig. 3:**
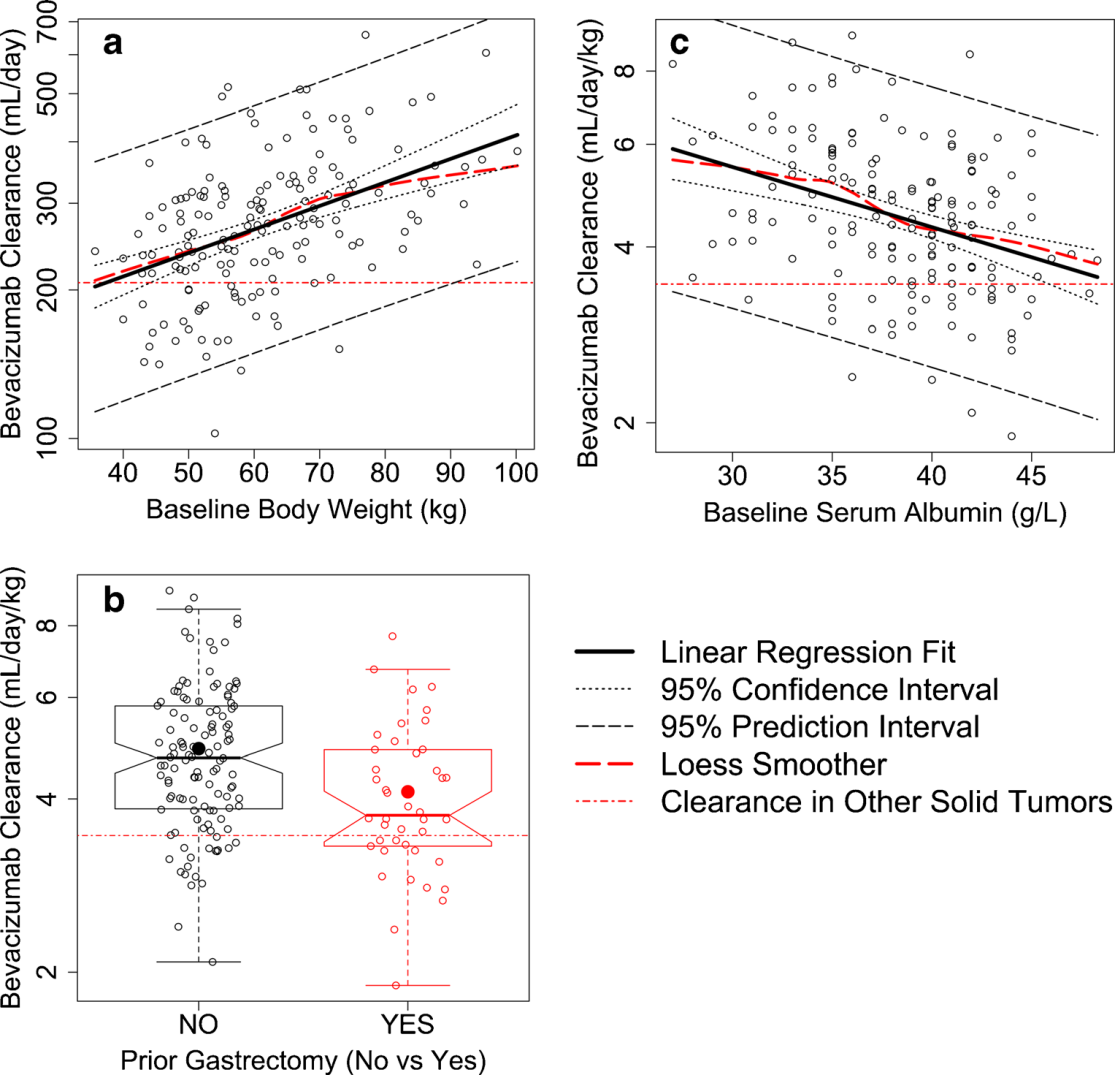
Significant correlation of bevacizumab clearance with patient variables **a** baseline body weight, **b** gastrectomy, and **c** baseline serum albumin. *Solid symbols* represent the mean. *Hollow symbols* represent individual data. The clearance in other solid tumors was calculated by taking into the account the covariate values in this study

Among all the patient variables (demographic, prognostic, and biochemical factors) evaluated, baseline body weight exhibited the most significant correlation with bevacizumab clearance (*p* < 0.0001, Fig. 3a). Bevacizumab clearance significantly increased with increasing body weight, and therefore, weight-normalized clearance was correlated to other patient variables instead of absolute clearance. Figure 3b, c demonstrated that bevacizumab clearance was significantly faster (*p* = 0.0014) in patients without prior gastrectomy (median = 4.72 mL/day/kg, *n* = 120) than those with gastrectomy (median = 3.75 mL/day/kg, *n* = 42) with a ratio of geometric means of 1.19 (95% CI = 1.07 to 1.32). Bevacizumab weight-normalized clearance significantly decreases with increasing baseline albumin (*p* < 0.0001), and appeared to be faster in patients with lower total protein levels, higher Eastern Cooperative Oncology Group (ECOG) scores, more metastatic sites, and poorer response (Supplement Figures), but these correlations did not reach statistical significance. No statistically significant correlation was observed between bevacizumab clearance and tumor location (gastroesophageal junction *versus* stomach), type of gastric cancer (diffuse, intestinal, or mixed), baseline plasma VEGF-A, baseline lactate dehydrogenase, liver metastases, HER2 status, or gender.

Comparison of relevant patient characteristics between the Asian and Non-Asian patients is summarized in Table II. Body weight was significantly lower (*p* < 0.0001) in Asian patients. No statistically significant difference was observed in other patient variables between Asian and Non-Asian patients.

**Table II:** Comparison of Patient Characteristics between Asian and Non-Asian Patients with Evaluable Pharmacokinetic Data

	Asian (*n* = 101)	Non-Asian (*n* = 61)
Age (years)	58 (28∼76)	58 (31∼81)
Body weight (kg)	55.1 (35.7∼95.4)	67 (40∼100.2)
Serum albumin (g/L)	38 (27∼47)	40 (29∼48.3)
Total protein (g/L)	68 (49∼81)	68 (56∼85.4)
Male/female	68.3%:31.7%	65.6%:34.4%
Gastrectomy (no/yes)	71.3%:28.7%	78.7%:21.3%
Number of metastatic sites >1	67.0%	74.6%
Sampling day after the last dose	26 (2∼99)	25 (3∼284)
Number of doses received	7 (1∼21)	8 (2∼21)

All values are expressed as median (range) or percentage of the patients

As shown in Fig. 1a, b, no significant separation of bevacizumab plasma concentrations was observed between Asian and Non-Asian patients. No significant pattern was observed between Asian and Non-Asian patients in term of the difference between observed and expected (simulated based on the reference PPK model) bevacizumab concentrations (Fig. 1c) and weight-normalized clearance (Fig. 2b).

**Fig. 4:**
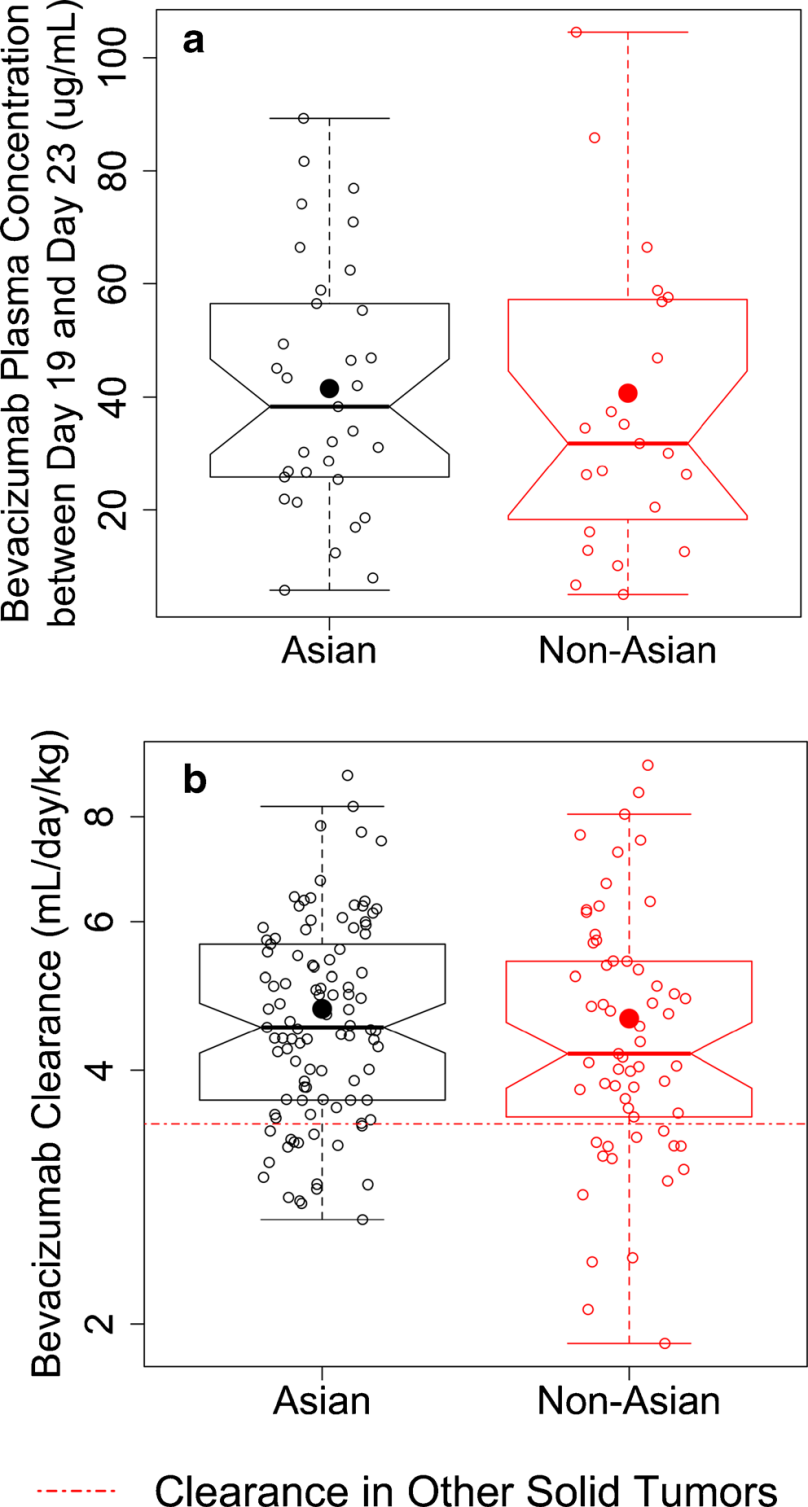
Comparison of bevacizumab exposure and clearance between Asian and Non-Asian patients **a** bevacizumab through concentrations collected between days 19 and 23 after the last dose and **b** bevacizumab clearance. *Solid symbols* represent the mean. *Hollow symbols* represent individual data. The clearance in other solid tumors was calculated by taking into the account the covariate values in this study

Statistical comparison of bevacizumab trough concentrations and clearance between Asian and Non-Asian AGC patients is summarized in Fig. 4. Due to the large variability in sampling time after the last dose of bevacizumab, only bevacizumab concentrations collected within a reasonable time window around the end of a dosing cycle (21 days after dose) were included in the analysis. Bevacizumab concentrations collected between day 19 and day 23 (*n* = 56) averaged 41.5 and 40.7 mcg/mL in Asian (*n* = 33) and Non-Asian (*n* = 23) patients, respectively, and no statistically significant difference was observed with a *p* value of 0.4761 (Fig. 4a). The ratio of geometric means of concentrations in Asian and Non-Asian patients was 1.16 (95% CI = 0.76 to 1.77). Altering the time window yielded similar results.

Weight-normalized bevacizumab clearance averaged 4.73 and 4.61 mL/day/kg in Asian (*n* = 101) and Non-Asian (*n* = 61) patients, respectively, and no statistically significant difference was observed with a *p* value of 0.3652 (Fig. 4b). The ratio of geometric means of concentrations in Asian and Non-Asian patients was 1.05 (95% CI = 0.95 to 1.16).

## DISCUSSION

This is the first study investigating bevacizumab pharmacokinetic behavior and exposure in advanced gastric cancer (AGC). This analysis attempted to address two critical questions: (1) is bevacizumab exposure and pharmacokinetics different in AGC and other solid tumors and (2) which patient variables (demographic, prognostic, and biochemical factors) influence bevacizumab exposure and pharmacokinetics in AGC? Our study demonstrated significantly lower bevacizumab pharmacokinetic exposure in patients with AGC and an approximate 50% increase in clearance *versus* other solid tumors, consistent with observations in the intravenous formulation of trastuzumab (18,19) and pertuzumab (21). Bevacizumab was also found to be cleared significantly faster in AGC patients without prior gastrectomy. Bevacizumab exposure and clearance was not significantly different between Asian and Non-Asian AGC patients.

To date, the existing reference population pharmacokinetic (PPK) model was the most comprehensive pharmacokinetic evaluation of bevacizumab in various solid tumors; however, AGC was not included in the PPK model, as clinical trials had not been performed in AGC by the time when the PPK model was developed. The PPK model contained data that represented both bevacizumab as a single agent or in combination with chemotherapy, both single- and multiple-dose administration, and both rich and sparse data. Therefore, it was an ideal reference population and model to evaluate the difference in bevacizumab pharmacokinetics between AGC and other solid tumors. The final model has incorporated all relevant patient variables that have a significant impact on bevacizumab pharmacokinetics, and therefore, eliminated the influence of the possible imbalance of patient characteristics between AGC patients in this analysis and the reference patient population (other solid tumors).

Two distinct approaches were employed to compare bevacizumab pharmacokinetics in AGC with other solid tumors. One approach was the visual predictive check (VPC) to compare observed bevacizumab concentrations in AGC patients against expected concentrations assuming the same bevacizumab pharmacokinetics in AGC as in other solid tumors (reference PPK model). The other approach was Empirical Bayes estimation to compare estimated bevacizumab pharmacokinetic parameters based on observed data against expected parameters assuming same bevacizumab pharmacokinetics in AGC as in other solid tumors.

The VPC showed strong evidence that AGC patients were underexposed to bevacizumab as compared to other solid tumors when given the same dose (Fig. 1). The observed bevacizumab concentrations were significantly lower than expected by 47% on average (*p* < 0.0001). The observed concentrations were below the expected concentrations in majority of the patients.

The Empirical Bayes estimation suggested that faster clearance is the potential cause of the bevacizumab underexposure in AGC. Median weight-normalized clearance was 4.5 mL/day/kg in AVAGAST, which is approximately 50% faster than other solid tumors (3 mL/day/kg). The estimated clearance based on observed AVAGAST data is significantly faster than expected (*p* < 0.0001, Fig. 2a), and is above expected in majority of the patients (Fig. 2b).

The limitation of Empirical Bayes estimation is that volume of distribution (Vd) cannot be reliably estimated due to the sparse sampling, and therefore, the variation in the observed concentrations is predominantly translated into the variation in clearance. Therefore, we could not reliably compare Vd in AGC against other solid tumor or associate Vd with ethnicity or patient variables in AGC. However, Vd of bevacizumab carries small inter-individual variability and is roughly equal to the plasma volume (15). Therefore, it is reasonable to focus on the clearance of bevacizumab.

The underlying mechanism for the lower exposure and faster clearance of bevacizumab in AGC is not clear. One possible hypothesis is that cancer cachexia may cause bevacizumab and IgG monoclonal antibody to be consumed as source of protein, especially considering that the gastrointestinal dysfunction in AGC patients may significantly reduce the absorption of nutrients. However, no cachexia was observed in the patient population in this analysis. Another possible hypothesis is that the gastric dysfunction and gastrectomy in AGC patients may alter IgG elimination *via* catabolic mechanisms or altered blood flow. Further research is warranted to investigate the underlying mechanism. Pharmacokinetic drug–drug interaction (DDI) between bevacizumab and cisplatin/capecitabine is unlikely the cause because such DDI was not observed in previous clinical trials (data unpublished). In general, small-molecule drugs (*e.g*., chemotherapy agents) and monoclonal antibodies (*e.g*., bevacizumab) do not share clearance pathways. Therefore, it is not expected that chemotherapy agents would have any potential interactions with or direct alterations of the clearance or exposure of monoclonal antibodies. Target-mediated disposition is also unlikely the cause because the molar concentration of bevacizumab is thousands of times higher than that of the target (VEGF-A), resulting in target saturation.

Our observation that baseline body weight and albumin are significantly associated with bevacizumab clearance in AGC is consistent with previous findings in other solid tumors (14,15). It is well known that clearance of other IgG antibodies is faster in patients with lower serum albumin levels (26). This effect may be due to the mechanism for albumin turnover, which is also mediated by FcRn (neonatal Fc receptor) recycling (27,28). Therefore, low albumin levels might reflect decreased efficiency of FcRn catabolic/recycling capability, which correlates to faster bevacizumab clearance leading to lower exposure.

Gastrectomy is a unique patient variable to AGC. It is well known that patients with prior gastrectomy have better clinical outcomes and better health status. Our analysis demonstrated that bevacizumab clearance was significantly faster (*p* = 0.0014) in patients without prior gastrectomy, likely because gastrectomy results in or indicates better patient health status, while it is well known that clearance of bevacizumab is slower in patients with better health status. Any other underlying mechanism responsible for faster bevacizumab clearance in patients without prior gastrectomy is unknown and warrants further research. Our study also demonstrated that bevacizumab clearance appeared to be faster in patients with lower total protein, higher ECOG scores, more metastatic sites and poorer response (Supplement Figures), but these correlations did not reach statistical significance, likely due to small sample size. All the significant correlations between bevacizumab clearance and patient variables were consistent when the base reference PPK model or the final covariate PPK model was used to estimate the clearance.

In recent several multi-regional studies of AGC, longer overall survival was observed in Asian patients, and hazard ratios of overall survival between the treatment and control arm were different in Asian and Non-Asian patients (Supplement Table 1). It was unknown whether these differences could result in different bevacizumab pharmacokinetics between Asian and Non-Asian patients, or *vice versa*. Our analysis did not demonstrate different bevacizumab exposure or clearance between Asian and Non-Asian patients (Figs. 1, 2b, and 4). Relevant patient characteristics were well balanced, especially all the variables that were found significantly correlated with weight-normalized clearance of bevacizumab (albumin and gastrectomy). It is worth mentioning that the absolute bevacizumab clearance (mL/day) was significantly lower in Asian AGC patients (*p* = 0.0134), but body weight was also significantly lower in Asian AGC patients (*p* < 0.0001), and no difference in weight-normalized clearance was observed between Asian and Non-Asian patients. This suggested that the difference in bevacizumab absolute clearance by ethnicity is most likely caused by the body weight difference between Asian and Non-Asian patients.

Our analysis has several limitations. First of all, there was only one sample from each patient, which made it impossible to perform comprehensive population pharmacokinetic modeling with covariate analysis. However, the two approaches we employed (VPC and Empirical Bayes estimation) successfully overcame this challenge of sparse sampling and adequately addressed the primary scientific questions (difference in bevacizumab pharmacokinetics between AGC and other solid tumors and between Asian and Non-Asian AGC patients). Secondly, the sample was taken at random times after the last dose of bevacizumab instead of nominal time, resulting in a large variability in sampling time. This made it challenging to compare bevacizumab concentrations between subgroups due to the decline of concentration over time. Fortunately, the actual time of sampling was recorded, and we were able to adjust for this variability in sampling time by only comparing bevacizumab concentrations collected within a relatively narrow time window around the end of a dosing cycle (21 days after dose), such as days 19 to 23, which is adequate given that bevacizumab has a long half life of 20 days. The pharmacokinetic analysis was also repeated in patients who received more than five or six doses at the time of sampling in order to make sure that the assumption of steady state is valid. The results are very similar to the current analysis and did not change the conclusion. Finally, the number of patients with evaluable pharmacokinetic samples was relatively small, partly due to the high death rate of AGC patients and partly due to the random sampling time as bevacizumab concentrations were below LLOQ in many samples that were collected too long after the last bevacizumab dose. However, all efforts were made to collect samples from all patients, and 47% of the patients in the bevacizumab arm were able to contribute samples.

In conclusion, AGC patients exhibited significantly lower bevacizumab exposure due to an approximate 50% increase in clearance *versus* other solid tumors, consistent with observations in the intravenous formulation of trastuzumab (18,19) and pertuzumab (21). Bevacizumab is cleared faster in patients without prior gastrectomy. No significant difference in bevacizumab pharmacokinetics was observed between Asian and Non-Asian patients. The low bevacizumab exposure in AGC could potentially fall below the drug exposure that has been proven efficacious in other solid tumors, especially considering that the lowest approved clinical dosing regimen for bevacizumab (7.5 mg/kg every 3 weeks) was used in AVAGAST. The underlying mechanism for faster bevacizumab clearance in AGC patients and especially in AGC patients without gastrectomy is unknown and warrants further research.

## Electronic Supplementary Material

Below is the link to the electronic supplementary material.

ESM 1 (DOC 2334 kb)
