# Supplementary material for: Lower Exposure and Faster Clearance of Bevacizumab in Gastric Cancer and the Impact of Patient Variables: Analysis of Individual Data from AVAGAST Phase III Trial
Source: AAPS J. 2014 Jun 19;16(5):1056–63. doi: 10.1208/s12248-014-9631-6 (PMC4147052; doi:10.1208/s12248-014-9631-6)

**Supplement Table 1: Different survival results between Eastern and Western patient populations in recent multi-regional large randomized double-blind Phase III trials**

| Study | Region | N | mOS in  Control arm  (Month) | mOS in  Study arm  (Month) | Hazard ratio  (95% CI) |
| --- | --- | --- | --- | --- | --- |
| AVAGAST ^13^ | Asia | 376 | 12.1 | 13.9 | 0.97 (0.75;1.25) |
|  | Pan-America | 149 | 6.8 | 11.5 | 0.63 (0.43;0.94) |
|  | Europe | 249 | 8.6 | 11.1 | 0.85 (0.63;1.14) |

mOS: median overall survival

N: number of patients

CI: confidence interval

**Supplement Table 2: Model parameters of final reference population PK model (PPK model) of other solid tumors**

The analysis included more than 5831 bevacizumab concentrations from 533 patients in eight clinical trials (Phase I, II and III) who received intravenous doses of bevacizumab ranging from 1 to 20 mg/kg at a dosing frequency ranging from every 1 to 3 weeks. Bevacizumab clearance was significantly associated with baseline body weight, albumin, total protein and gender. Central volume of distribution (V1) was significantly associated with body weight, gender, tumor burden and albumin. Peripheral volume of distribution (V2) was significantly associated with concomitant chemotherapy and age.

Between-subject variability model:

P_j_ = TVP * exp(η_j_), where TVP is the typical value of the pharmacokinetic parameter clearance (CL), central volume of distribution (V1) and peripheral volume of distribution (V2) in the population, P_j_ is the individual value for P in the *jth* individual, and η_j_ is a random variable. Etas (η_j_) are independent, identically and symmetrically distributed errors with mean zero and variance ω_P_^2^.

Residual variability model (a proportional error model): C_obs,ij_ = C_pred, ij_ · (1 + ε_ij 1_), where C_obs,ij_ is the i^th^ plasma concentration measured in the j^th^ patient, C_pred, ij_ is the respective model predicted concentration, and the ε_ij 1_ is unobserved random variable. The ε_ij 1_ is independent, symmetrically and not identically distributed with mean zero and a variance equal to σ^2^.

For clearance:

$$TVCL=\theta_{1}+\theta_{6}\cdot SEX+\theta_{7}\cdot\left(WGT-74.5\right)+\theta_{8}\cdot\left(TPRO-70\right)+\theta_{9}\cdot\left(ALBU-37\right)$$

For central volume:

$$TVV1=\theta_{1}+\theta_{10}\cdot SEX+\theta_{11}\cdot\left(WGT-74.5\right)+\theta_{12}\cdot\left(ALBU-37\right)+\theta_{13}\cdot\left(BURM-18.8\right)$$

For peripheral volume:

$$TVV2=\theta_{1}+\theta_{14}\cdot CHEMO+\theta_{15}\cdot\left(AGE-59\right)$$

| **Parameter** | **Estimate** |
| --- | --- |
| ***Fixed Effect Parameters*** |  |
| θ_1_ Clearance (CL, mL/h) | 8.58 |
| θ_2_ Central Volume (V1, mL) | 2660 |
| θ_3_ Inter-compartmental clearance (Q, mL/h) | 30.5 |
| θ_4_ Peripheral Volume (V2, mL) | 2160 |
| θ_6_ Gender on CL (SEX) | 2.23 |
| θ_7_ Weight on CL (WGT, kg) | 0.0422 |
| θ_8_ Total protein on CL (TPRO, g/L) | 0.0767 |
| θ_9_ Albumin on CL (ALBU, g/L) | -0.345 |
| θ_10_ Gender on V1 (SEX) | 498 |
| θ_11_ Weight on V1 (WGT, kg) | 17.3 |
| θ_12_ Albumin on V1 (ALBU, g/L) | -15.4 |
| θ_13_ Tumor burden on V1 (BURM, cm^2^) | 2.37 |
| θ_14_ Chemotherapy on V2 | 1660 |
| θ_15_ Age on V2 (AGE, years) | 25.5 |
| ***Random Effect Parameters*** (% CV) |  |
| ω_1_^2^ BSV on CL | 25.7 |
| ω_2_^2^ BSV on V1 | 14.4 |
| ω_3_^2^ BSV on V2 | 43.1 |
| θ_5_ Proportional residual error | 18.7 |

SEX, binary covariate for gender: SEX=0 for female patients / SEX=1 for male patients

CHEMO, binary covariate for chemotherapy: CHEMO=0 when bevacizumab is administered alone / CHEMO=1 when bevacizumab is co-administered with chemotherapy agents

**Supplement Figures** Correlation between bevacizumab clearance and patient variables that demonstrated trend but did not reach statistical significance. CR, complete response; PD, progressive disease; PR: partial response; SD, stable disease. Solid symbols represent the mean. Hollow symbols represent individual data. The clearance in other solid tumor was calculated by taking into the account the covariate values in this study.


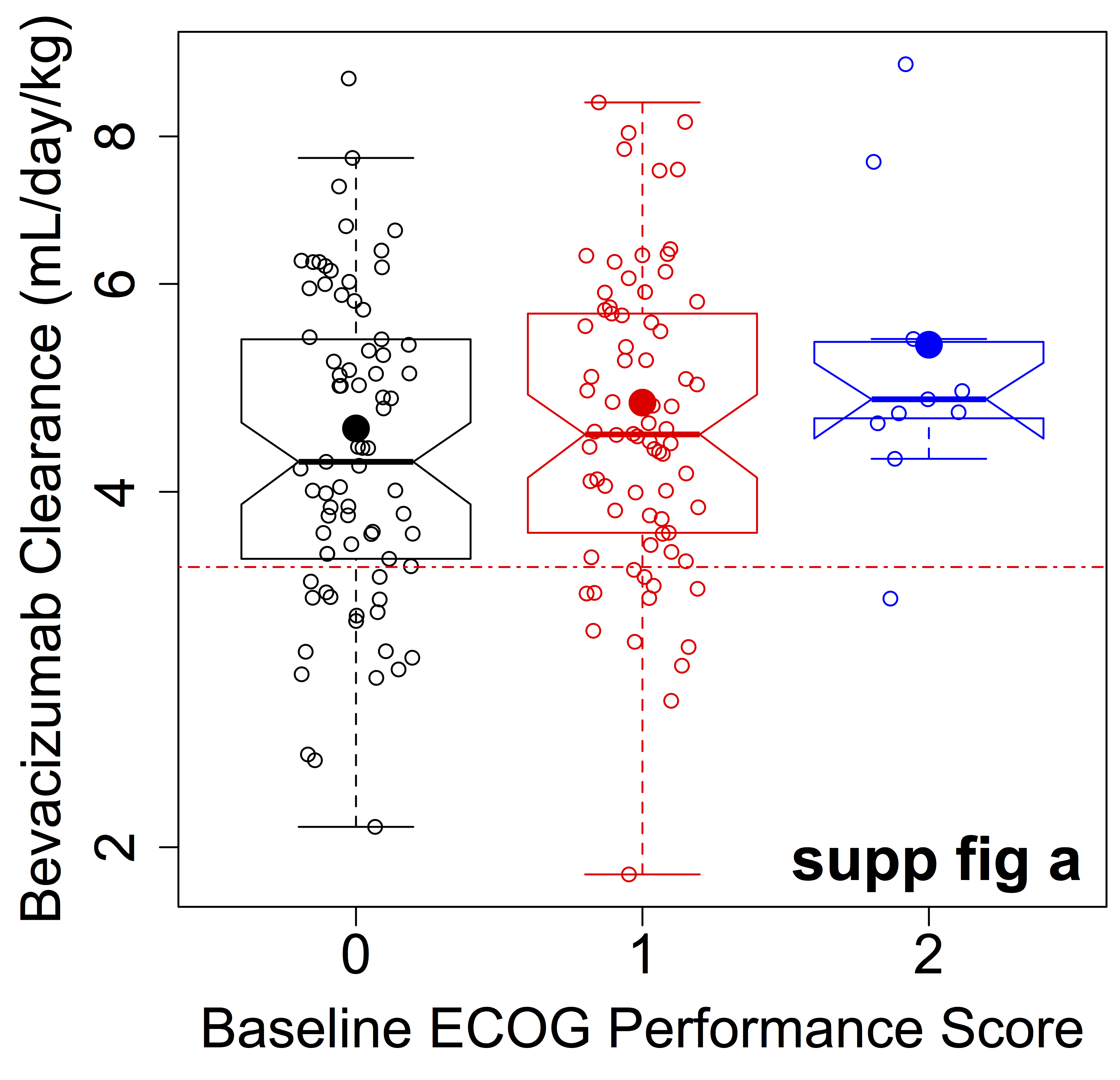


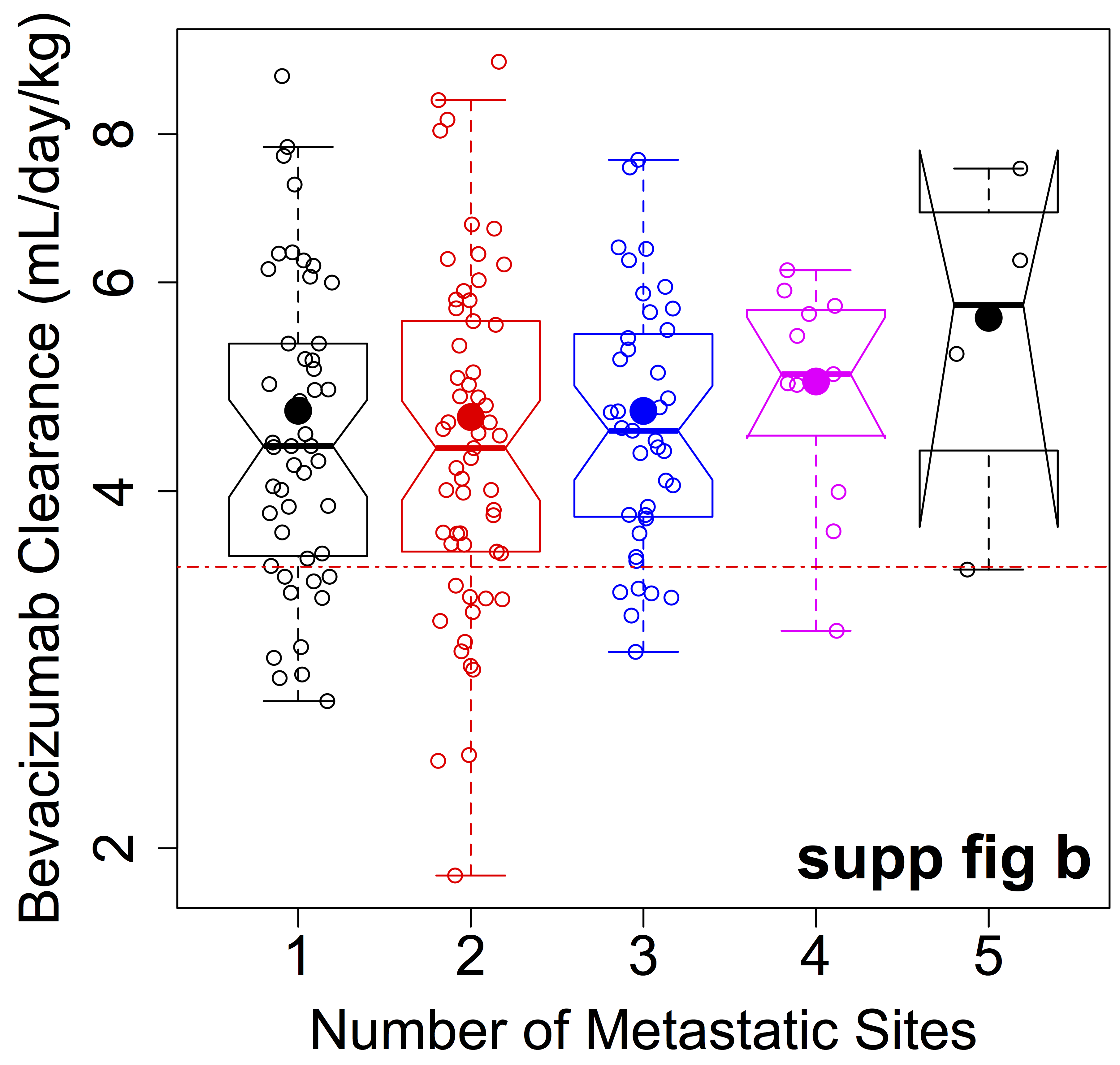


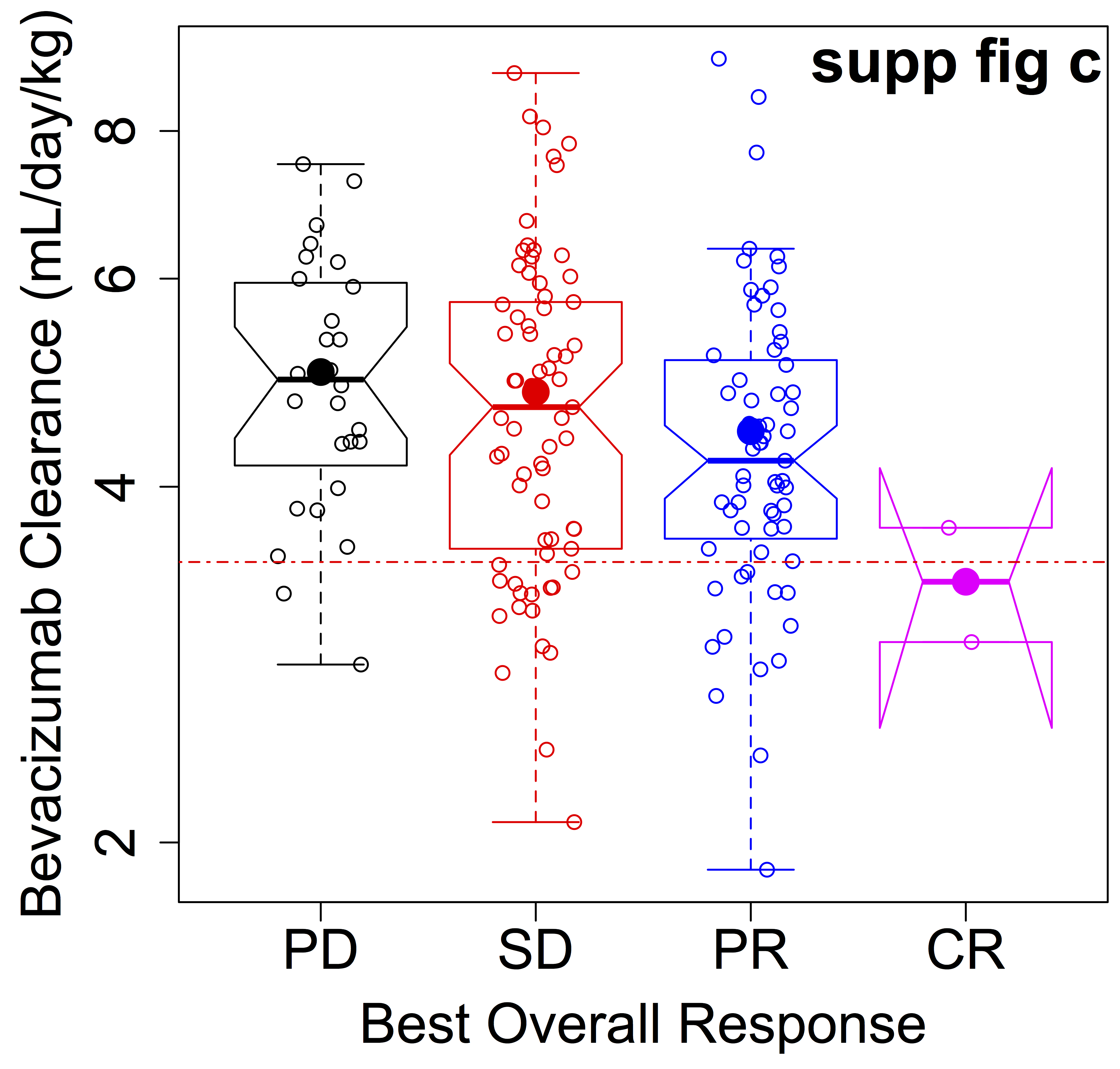


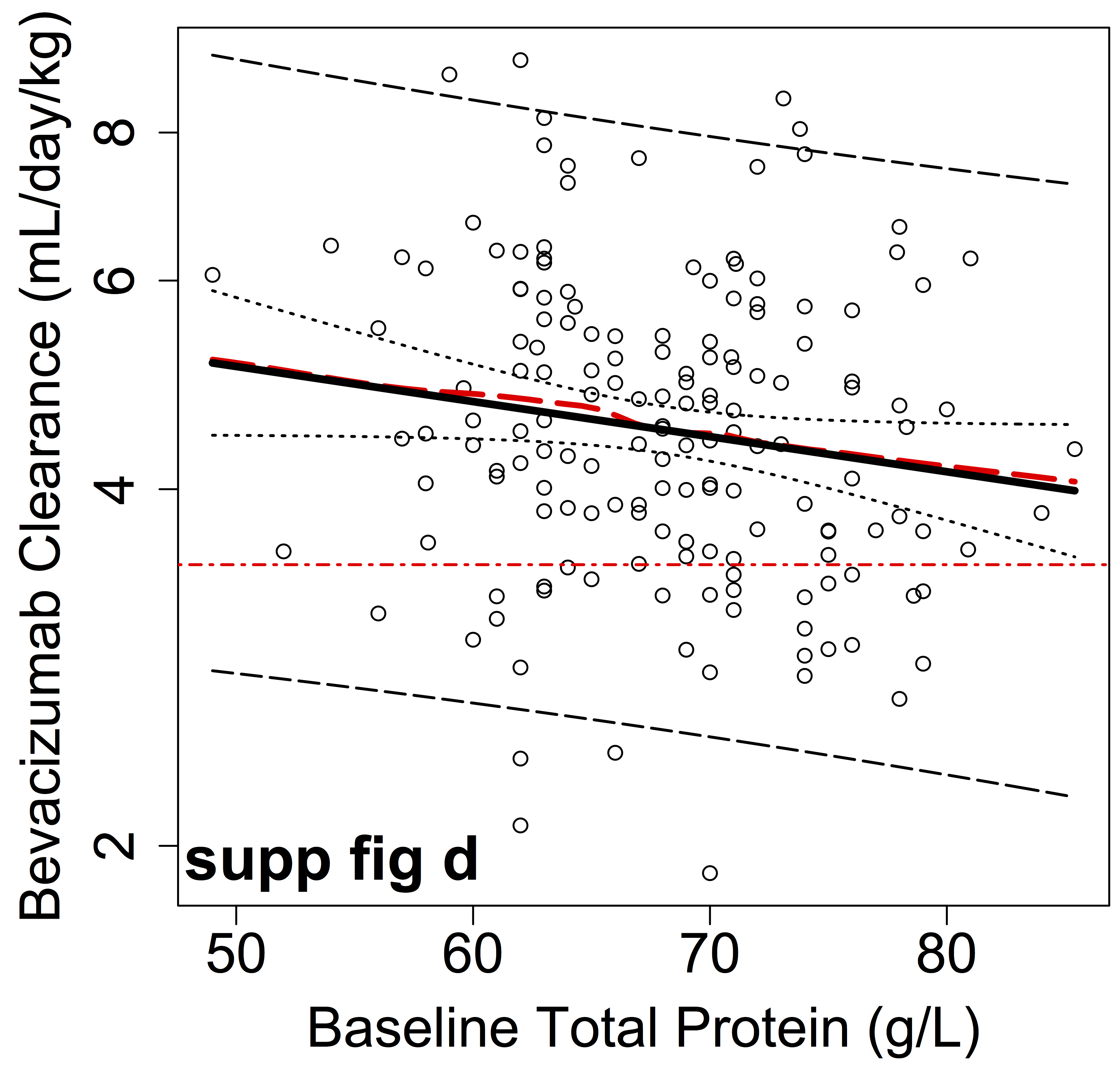


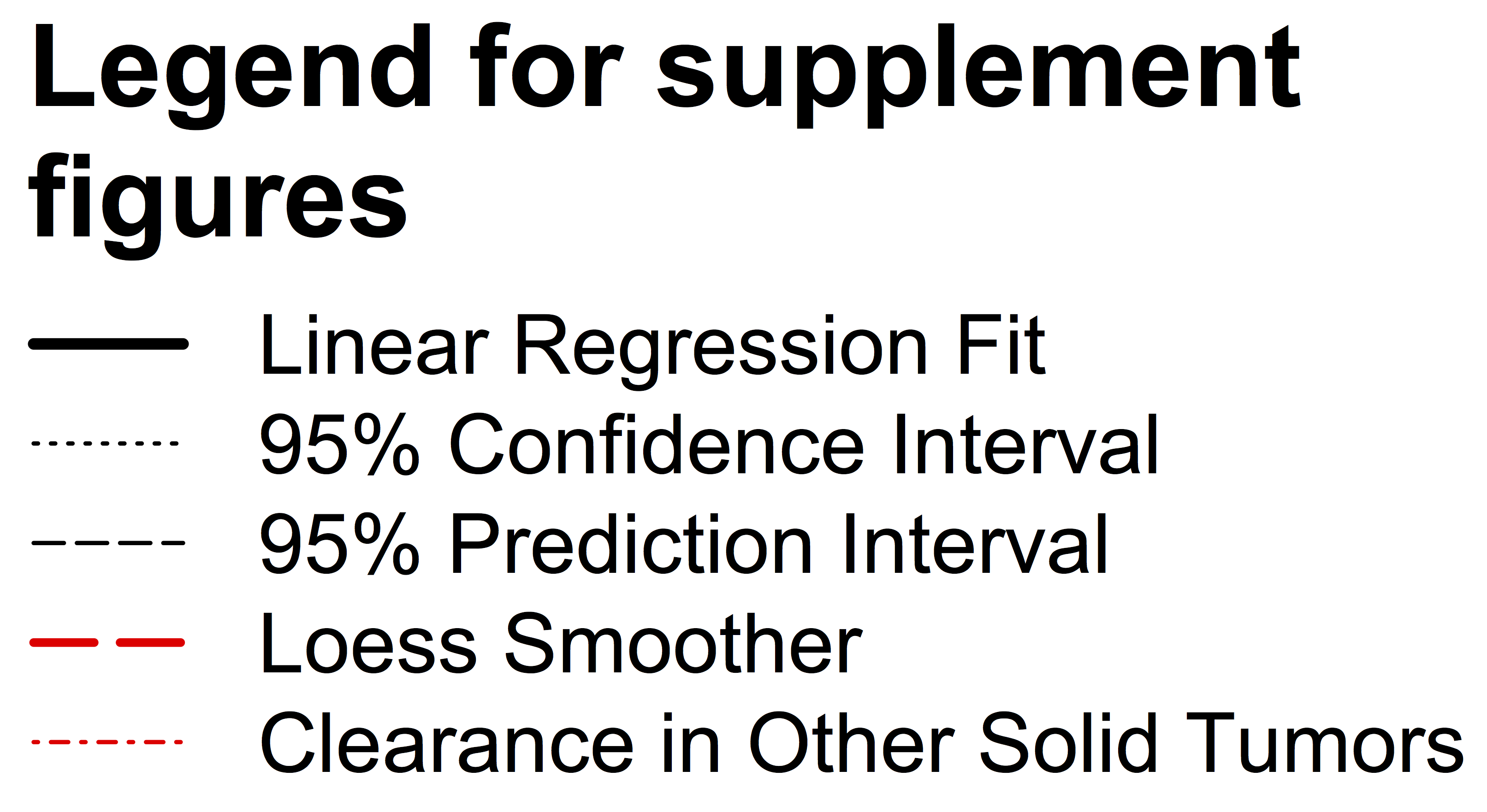

Supplement: Supplementary file 1 — (DOC 2334 kb) [file 12248_2014_9631_MOESM1_ESM.doc]
